# Identification of Differential Responses of Goat PBMCs to PPRV Virulence Using a Multi-Omics Approach

**DOI:** 10.3389/fimmu.2021.745315

**Published:** 2021-10-04

**Authors:** Roger-Junior Eloiflin, Gaël Auray, Sylvie Python, Valérie Rodrigues, Martial Seveno, Serge Urbach, Khadija El Koulali, Philippe Holzmuller, Philippe Totte, Genevieve Libeau, Arnaud Bataille, Artur Summerfield

**Affiliations:** ^1^ CIRAD (Agricultural Research Centre for International Development), UMR (Unité Mixte de Recherche), ASTRE (Animal, Health, Territories, Risks and Ecosystems), Montpellier, France; ^2^ ASTRE (Animal, Health, Territories, Risks and Ecosystems), University of Montpellier, CIRAD (Agricultural Research Centre for International Development), INRAE (Institut National de Recherche pour l'Agriculture, l'Alimentation et l'Environnement), Montpellier, France; ^3^ Institute of Virology and Immunology, Mittelhäusern, Switzerland; ^4^ Department of Infectious Diseases and Pathobiology, Vetsuisse Faculty, University of Bern, Bern, Switzerland; ^5^ CIRAD (Agricultural Research Centre for International Development), UMR (Unité Mixte de Recherche), ASTRE (Animal, Health, Territories, Risks and Ecosystems), Petit-Bourg, France; ^6^ BCM (BioCampus Montpellier), Univ. Montpellier, CNRS (Centre national de la recherche scientifique), INSERM, Montpellier, France; ^7^ IGF (Institut de Génomique Fonctionnelle), Univ. Montpellier, CNRS (Centre national de la recherche scientifique), INSERM, Montpellier, France

**Keywords:** peste des petit ruminant virus, PBMC, analysis, virulence, saanen goat

## Abstract

Peste des petits ruminants (PPR) is an acute transboundary infectious viral disease of small ruminants, mainly sheep and goats. Host susceptibility varies considerably depending on the PPR virus (PPRV) strain, the host species and breed. The effect of strains with different levels of virulence on the modulation of the immune system has not been thoroughly compared in an experimental setting so far. In this study, we used a multi-omics approach to investigate the host cellular factors involved in different infection phenotypes. Peripheral blood mononuclear cells (PBMCs) from Saanen goats were activated with a T-cell mitogen and infected with PPRV strains of different virulence: Morocco 2008 (high virulence), Ivory Coast 1989 (low virulence) and Nigeria 75/1 (live attenuated vaccine strain). Our results showed that the highly virulent strain replicated better than the other two in PBMCs and rapidly induced cell death and a stronger inhibition of lymphocyte proliferation. However, all the strains affected lymphocyte proliferation and induced upregulation of key antiviral genes and proteins, meaning a classical antiviral response is orchestrated regardless of the virulence of the PPRV strain. On the other hand, the highly virulent strain induced stronger inflammatory responses and activated more genes related to lymphocyte migration and recruitment, and inflammatory processes. Both transcriptomic and proteomic approaches were successful in detecting viral and antiviral effectors under all conditions. The present work identified key immunological factors related to PPRV virulence *in vitro*.

## Introduction

Peste des petits ruminants (PPR) is an acute transboundary infectious viral disease of small ruminants, mainly sheep and goats which threatens the livelihoods and food security of smallholder farmers across Africa, the Middle East and Asia (1, 2). Recent outbreaks in Georgia, Bulgaria and the current distribution of the disease point to potential risks to Europe (3–5). Wild and captive artiodactyls, cattle, camelids and suids can also be infected by the disease (6, 7). The causal agent of this disease is the peste des petits ruminants virus (PPRV) (family: *Paramyxoviridea*, genus: *Morbillivirus*). Genotypic classification of PPRV has identified four lineages. These lineages are all circulating in Africa (8) while lineages III and IV are circulating in Middle East (9) and lineage IV in Asia (10) with a recent incursion in Europe (11).

Infected animals are characterised by marked hyperthermia, apathy, anorexia, diarrhoea, ocular and nasal discharge, and lip and nose lesions. Mortality can reach 50-100% in naïve small ruminant populations. Due to the impact of the disease on food security and on the global economy, the OIE and FAO launched a worldwide campaign to eradicate PPRV by 2030 (12). Such an eradication programme requires effective vaccines and diagnostic tests. Two live attenuated PPRV vaccines, Nigeria 75/1 and Sungri96, are currently in use worldwide. Both vaccines provide complete clinical protection against all four PPRV lineages (13), irrespective of whether they are administered intranasally or subcutaneously (14). One dose of Nigeria75/1 vaccine (10^2.5^ TCID50) has been shown to induce protective immunity for at least 3 years in vaccinated animals (15). Host susceptibility varies considerably depending on the PPRV strain, and on the species and breed of the host (16, 17). Identifying factors that influence host-pathogen interactions and the outcome of an infection may greatly help develop efficient PPR control strategies.

Like other morbilliviruses, PPRV is lymphotropic and epitheliotropic. PPRV infection is associated with immunosuppression, as indicated by leukopenia and lymphopenia, providing a window of opportunity for the virus to replicate and spread (18). This phenomenon appears to be a multifactorial process involving interactions between PPRV haemagglutinin H and the cellular receptors (PRR and SLAM) and the suppression of the interferon antiviral response by the non-structural viral proteins V and C (19, 20). Vaccination with live attenuated vaccines may also cause transient leukopenia without significantly affecting the immune response (21, 22).

Immune cells (lymphocytes, monocytes and dendritic cells) are responsible for transport of the virus to the tonsillar tissue and lymph nodes draining the initial site of infection where extensive viral replication occurs before propagation to epithelial and lymphoid tissues of other organs (23). In-vitro studies using peripheral blood mononuclear cells (PBMCs) have shed some light on the immune response to PPRV infections (24–26). In PBMCs infected with PPRV apoptotic indices were found including peripheral condensation of chromatin, nucleus and cell fragmentation leading to the formation of apoptotic bodies (27). These findings suggest that PPRV can induce apoptosis in goat lymphocytes. Transcriptome analysis of PBMCs infected by the PPRV vaccine strain Sungri/96 showed the dysregulation of both immune regulatory pathways and the expression of genes encoding the transcription factors that govern the response to viral infection (24). Innate immune molecules and antiviral genes seem to play an important role at the earliest state in infection of PBMCs, suggesting a link between innate and adaptive immune response to PPRV infection (25, 26).

In-vitro studies have generally only focussed on either a vaccine strain or highly virulent strains. The effect of strains with different levels of virulence on the modulation of the immune system has not been thoroughly compared in an experimental setting so far. Furthermore, gene expression studies of immune response to PPR have never been combined with protein expression analyses to confirm patterns of immune responses, except for the modulation of microRNA expression (28, 29). Multi-omics approaches are now favoured to increase our understanding of complex biological processes (30).

Reliable and reproducible challenge models to study host-PPRV interactions are needed for in-depth investigation of host-PPR interactions. In a previous study by our team, a model of PPRV challenge in Saanen goats was developed to test PPR vaccine efficacy (31). In that experiment, two virulent strains of PPRV were used to infect Saanen goats. In the Saanen goat breed, the Morocco 2008 PPRV strain was highly virulent, while the Ivory Coast 1989 PPRV strain only induced mild symptoms of the disease. No symptoms or virus shedding were observed with the PPRV vaccine strain Nigeria 75/1. In the present study, we use an in-vitro approach to explore the mechanisms behind the different levels of virulence of these three PPRV strains in the new challenge model.

In this study, we infected PBMCs from Saanen goats with the two virulent strains, Morocco 2008 and Ivory Coast 1989, and also with the vaccine strain Nigeria 75/1, the most commonly used PPRV vaccine strain (32). PPRV is known to interact with the signalling lymphocyte activation molecule (SLAM) (33). The mitogen Concanavalin A (ConA) was used to enhance virus susceptibility through enhanced SLAM expression and T cell activation. The level of SLAM expression in ConA-stimulated PBMCs is higher than in unstimulated cells and thus increases viral replication and titer (34). In this favourable context, PPRV strains have an optimal level of infectivity and inhibit lymphoblastogenesis in a dose-dependent manner (35). In the present study, we explored the response of PBMCs to infection by PPRV strains of different virulence using flow cytometry, transcriptomic analyses and proteomic analyses.

## Materials and Methods

### Ethics Statement

Five adult Saanen goats (males, castrated) kept at the Institute of Virology and Immunology IVI in Mittelhäusern, Switzerland served as blood donors. Blood sampling was performed in compliance with the Swiss animal protection law (TSchG SR 455; TSchV SR 455.1; TVV SR 455.163) under the authorization number BE8/19. The experiments were reviewed by the cantonal committee on animal experiments of the canton of Bern, Switzerland, and approved by the cantonal veterinary authority (Amt für Landwirtschaft und Natur LANAT, Veterinärdienst VeD, Bern, Switzerland).

### PBMC Isolation, Proliferation Assay and Virus Infection

Goat PBMCs were isolated from whole blood (n = 5 healthy adult Saanen goat donors) by gradient density separation using Histopaque-1077 (Sigma-Aldrich) according to the manufacturer’s instructions. Isolated PBMCs from each goat were divided into two sets of cultures.

Cell trace violet-labelled (see below) PBMCs were used for flow cytometry-based proliferation analysis, while unlabelled cells were used for RNA sequencing and proteomic analysis. PBMCs were cultured in DMEM supplemented with 10% foetal bovine serum, 1% non-essential amino acids, 1% sodium pyruvate, 1% Hepes and 0.02 mM beta-mercaptoethanol (all components were purchased from ThermoFisher Scientific).

The highly virulent PPRV strain Morocco 2008 (PPRV MA08 VSD2 Vero 4 VSD1, lineage IV), the weakly virulent PPRV strain Ivory Coast 1989 (PPRV IC89 PM3 Vero 5, lineage I), and vaccine PPRV strain Nigeria 75/1 (PPRV N75/1, Cirad Master seed batch LK1 BK1 Vero 75, lineage II) were used to infect PBMCs. The 50% tissue culture infective dose per ml (TCID50/ml) was 10^3.7^ TCID50/ml for PPRV MA08, 10^5.0^ TCID50/ml for PPRV IC89 and 10^5.6^ TCID50/ml for PPRV N75/1.

For virus infection, labelled and unlabelled goat PBMCs were seeded in 96-well plates at a density of 2.5 10^5^ cells per well and inoculated with the different strains at a multiplicity of infection (MOI) of 0.001. PBMCs from different donors were distributed and then infected in different plates. After 1 h of absorption, infected PBMCs were washed with 1X PBS and maintained in DMEM supplemented with 2% of FBS and Concanavalin A (ConA, 2.5 µg/ml, Sigma Aldrich). Mock-infected PBMCs underwent the same treatment and were used as controls. A pool of three culture replicates of PPRV- and mock-infected cultures of PBMCs was harvested at 24, 48, 72, 96 and 120 hours post infection (hpi).

### Flow Cytometry Assay

A cell proliferation assay was performed using the CellTrace Violet Cell Proliferation Kit (ThermoFisher Scientific) according to the manufacturer’s instructions. Infected PBMCs were analysed by flow cytometry (BD FACSCanto II Cell Analyzer, BD Biosciences) using an anti-PPRV-N-FITC monoclonal antibody (clone 38-4, CIRAD, Montpellier, France). PBMCs were washed and 7AAD (Biolegend) was added in the dark 10 min prior to acquisition to assess the percentage of live cells. For the intracellular staining of the virus, cells were fixed in 4% paraformaldehyde (PFA) and permeabilised in 1X PBS supplemented with 0.1% of Saponin. Fixed and permeabilised cells were stained with a FITC-conjugated monoclonal antibody against PPRV N protein (1:100) diluted in 1X PBS supplemented with 0.3% of Saponin.

### RNA Isolation and RNA Library Construction

Total RNA was extracted from goat PBMCs using TRIzol (Invitrogen). Briefly, PBMCs were harvested in TRIzol (Life Technologies), and Chloroform : IAA (49:1, Sigma-Aldrich) was added to each tube. After a centrifugation step at 12,000g at 4°C for 15 min, the upper aqueous phase was transferred to a fresh tube containing 0.5 ml of 75% ethanol. After 10 minutes incubation at room temperature, the nucleic acids in 75% ethanol were loaded onto a NucleoSpin RNA column placed in a collection tube. The remaining RNA isolation steps were performed using the NucleoSpin RNA kit (Macherey Nagel) according to the manufacturer’s instructions. Total RNA quality and concentration were measured and the library prepared at the Next Generation Sequencing platform of the University of Bern, Switzerland. Only samples with RNA Quality Number (RQN) score ≥ 8 were sequenced on a NovaSeq 6000 instrument using an SP flow cell.

### RNA Seq Data Processing

Raw sequencing reads from each library were aligned with the *Capra hircus* Ensembl genome (ARS1 GCF_001704415.1) and the PPRV genome (GCF_000866445.1_ViralProj15499) using Hisat2 (v.2.1.0). Read count data were obtained using StringTie (v.2.0.3) and were imported in RStudio software (v.1.1.456) using the tximport package (v.1.20.0). Differential expression analysis between PPRV-infected and not-infected PBMCs was performed using the DESeq2 package. A false discovery rate (FDR) < 0.05 and a |log2 Fold Change| > 1 were set as the default thresholds for significant differential expression. Venn diagrams between up- and downregulated genes were drawn with InteractiVenn (accessible at http://www.interactivenn.net/). Gene ontology categories based on biological processes, molecular functions and cellular components were retrieved for all up- and down-regulated genes using gProfiler (36).

Ingenuity Pathways Knowledge Base in QIAGEN’s IPA software (QIAGEN, Redwood City, USA) was used with the lists of differentially expressed genes to identify the canonical pathways, upstream regulators and the most significant biological processes. Core and multiple analyses were performed for each dataset to identify the activated (Z score > 2) or inactivated (Z score < −2) canonical pathways and bio functions.

DEG lists were also ranked based on the P-values adjusted (FDR) for a gene set enrichment analysis with goat blood transcriptional modules (BTM). BTM represent gene sets created based on a high level of interactions identified *ex vivo* in human PBMC (37), we had previously adapted for use in sheep (38) and pigs (39). The annotation of these genes was based on either the gene descriptions given in the ENSMBL data based or on homology with other species, including cattle, pigs, human and/or mouse. Homologies were obtained using the Biomart software accessible on the ENSEMBL web site. The goat BTMs were adapted to the goat by replacing human genes with their goat homologs. This step required extensive manual curation for genes that differed in name or have not yet been annotated in goats. The homologies, novel annotations, and the final lists of BTM genes are provided in **Supplementary Materials**. Module activity during the different infections was compared using a false discovery rate < 0.01. This threshold was set to select specific BTMs and to reduce false positive BTMs.

### Proteomic Data Acquisition and Processing

PPRV-infected and not-infected PBMCs were prepared for proteome analysis using a homemade protocol. First PBMCs were washed twice with 1X PBS and collected in DIGE buffer at different times. DIGE buffer comprised 7 M urea, 2 M thiourea, 4% 3-[(3-cholamidopropyl)-dimethylammonio]-1-propanesulfonate (CHAPS) and 30 mM TrisHCL. The pH was adjusted to 8 with HCl. This step allowed cell lysis and the conservation of protein structure. The quantity of proteins obtained was evaluated using a 2D gel and colorimetric dosage.

Samples were transferred to the Functional Proteomics Platform (FPP, Montpellier, France) where tryptic digestion of the samples was carried out on S-Trap Mini Spin columns (Protifi). Samples were loaded onto a 50-cm reversed-phase column (75 mm internal diameter; Acclaim PepMap 100 C18; Thermo Fisher Scientific) and separated with an UltiMate 3000 RSLC system (Thermo Fisher Scientific) coupled to a QExactive HF system (Thermo Fisher Scientific). Tandem mass spectrometry analyses were performed in a data-dependent mode. Full scans (375–1,500 m/z) were acquired in the Orbitrap mass analyser with a resolution of 60,000 at 200 m/z. For the full scans, 3 × 10^6^ ions were accumulated within a maximum injection time of 60 ms. The 12 most intense ions with charge states ≥2 were sequentially isolated (1 × 105) with a maximum injection time of 100 ms and fragmented by higher-energy collisional dissociation in the collision cell (normalised collision energy of 28%) and detected in the Orbitrap analyser at a resolution of 30,000.

Raw spectra were processed with MaxQuant (v 1.6.10.43) (40) using standard parameters with label-free quantification (LFQ), iBAQ and matched between run options using Andromeda (41). Tandem mass spectrometry spectra were matched against the UniProt reference proteomes (release 2020_04; http://www.uniprot.org) of *C. hircus* and Peste-des-petits-ruminants virus (proteome IDs UP000291000 and UP000118493, respectively) and 250 frequently observed contaminants, as well as reversed sequences of all entries. The maximum false peptide and protein discovery rate was set at 0.01. The representative protein ID in each protein group was automatically selected using our in-house Leading tool (v3.4) (42). The protein-protein interaction was predicted using the STRING database (STRING: functional protein association networks, accessible at string-db.org) with *Capra hircus* as the reference species (43).

### Statistical Analysis

Differences in survival between PPRV-infected and not-PPRV-infected PBMCs at different times were analysed using two-way ANOVA. Differences between goat proteins detected by proteomics were analysed using Student’s t-test. Statistical tests were carried out with graphpadprism (v7) and Perseus (v1.6.10.43) on LFQ data (44).

## Results

### PPRV Infection Induces a Decline in Viability and Cell Division in PBMCs

**Figure 1:**
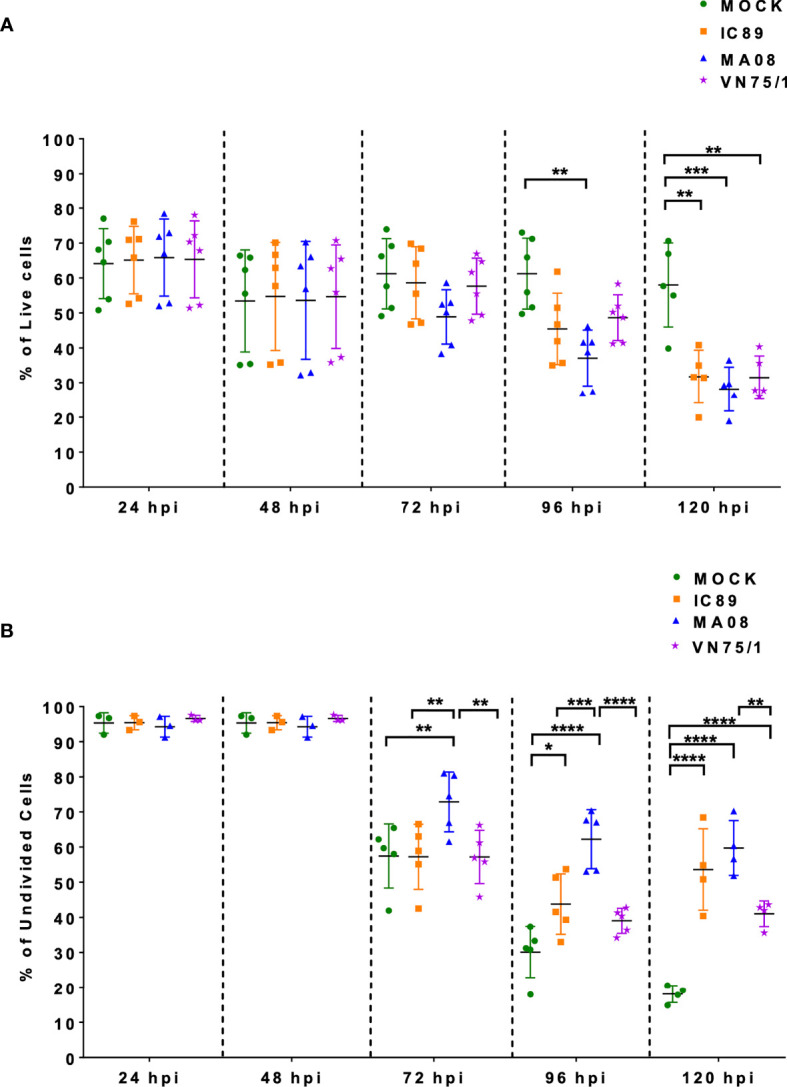
Effect of PPRV strains on ConA stimulated PBMC survival and proliferation over a period of five days. **(A)** Viability of the cells during infection expressed as the percentage of live cells (7AAD negative cells). **(B)** Frequencies of undivided cells after infection with the different virus strains. Five goats were used to obtain PBMCs. Goat PBMCs were infected with PPRV strains IC89, MA08 and VN75/1 at MOI of 0.001. The sixth point observed in 1A represents an additional experiment performed on one animal to confirm the results obtained. P-values were calculated using two-way ANOVA: “*” = P-values < 0.05; “**” = P-values < 0.01; “***” = P-values < 0.001; “****” = P-values < 0.0001.

Survival and proliferation of ConA stimulated PBMCs in response to infection with different PPRV strains were assessed by flow cytometry. Cell viability significantly decreased at 96 hours post-infection (hpi) after infection with MA08 compared to mock-infected cells. This significant effect was also observed with all PPRV strains at 120 hpi (**Figure 1A**). ConA stimulation induced multiple divisions of PBMCs. During the experiment, the proportion of undivided cells decreased from 100% to 20% in mock-infected PBMCs, from 100% to 50% in PBMCs infected with IC89, from 100% to 60% in PBMCs infected with MA08 and from 100% to 40% in PBMCs infected with VN751. This decrease started to significantly differ from that in the controls at 72 hpi when PBMCs were infected with MA08, at 96 hpi with IC89 and at 120 hpi with VN751 (**Figure 1B**).

### Viral Replication in PBMCs Differs Among PPRV Strains

**Figure 2:**
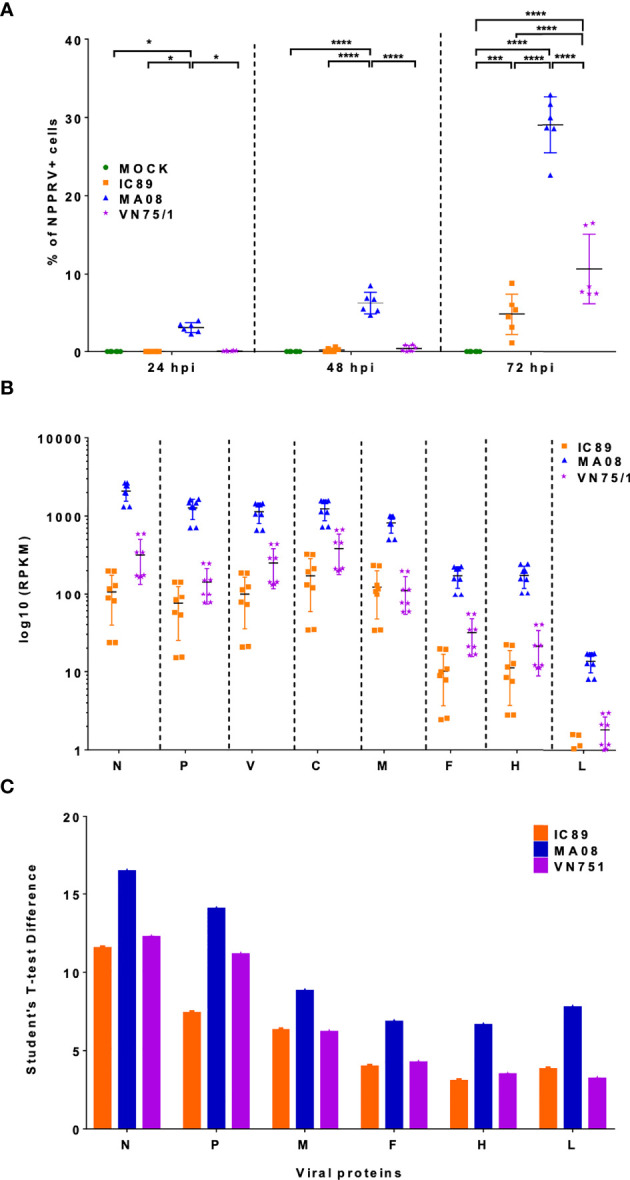
Detection of viral genes and proteins during PPRV infections of PBMCs. **(A)** Detection of PPRV N-nucleoprotein positive cells by flow cytometry. P-values were calculated using two-way ANOVA. **(B)** Distribution of mapped reads of viral genes in RNA sequencing data at 72 hpi. **(C)** Viral proteins accumulated at 72 hpi and detected by mass spectrometry. Five goats were used to obtain PBMCs. The sixth point observed in 2A represents an additional experiment performed on one animal to confirm the results obtained. P-values were calculated using two-way ANOVA: “*” = P-values < 0.05; “***” = P-values < 0.001; “****” = P-values < 0.0001.

The level of infection in PBMCs was first determined by staining for the intracellular presence of PPRV N. In ConA-stimulated PBMCs, MA08 showed higher infectivity over time compared to IC89 and VN751 (**Figure 2A**). This was linked with higher levels of mRNA in many viral genes (N, P, V, C, M, F, H, and L) at 72 hpi quantified by RNA sequencing (**Figure 2B**) and viral proteins quantified by mass spectrometry (**Figure 2C**).

### More Genes Are Differentially Regulated During Infection With the Highly Virulent PPRV Strain

**Figure 3:**
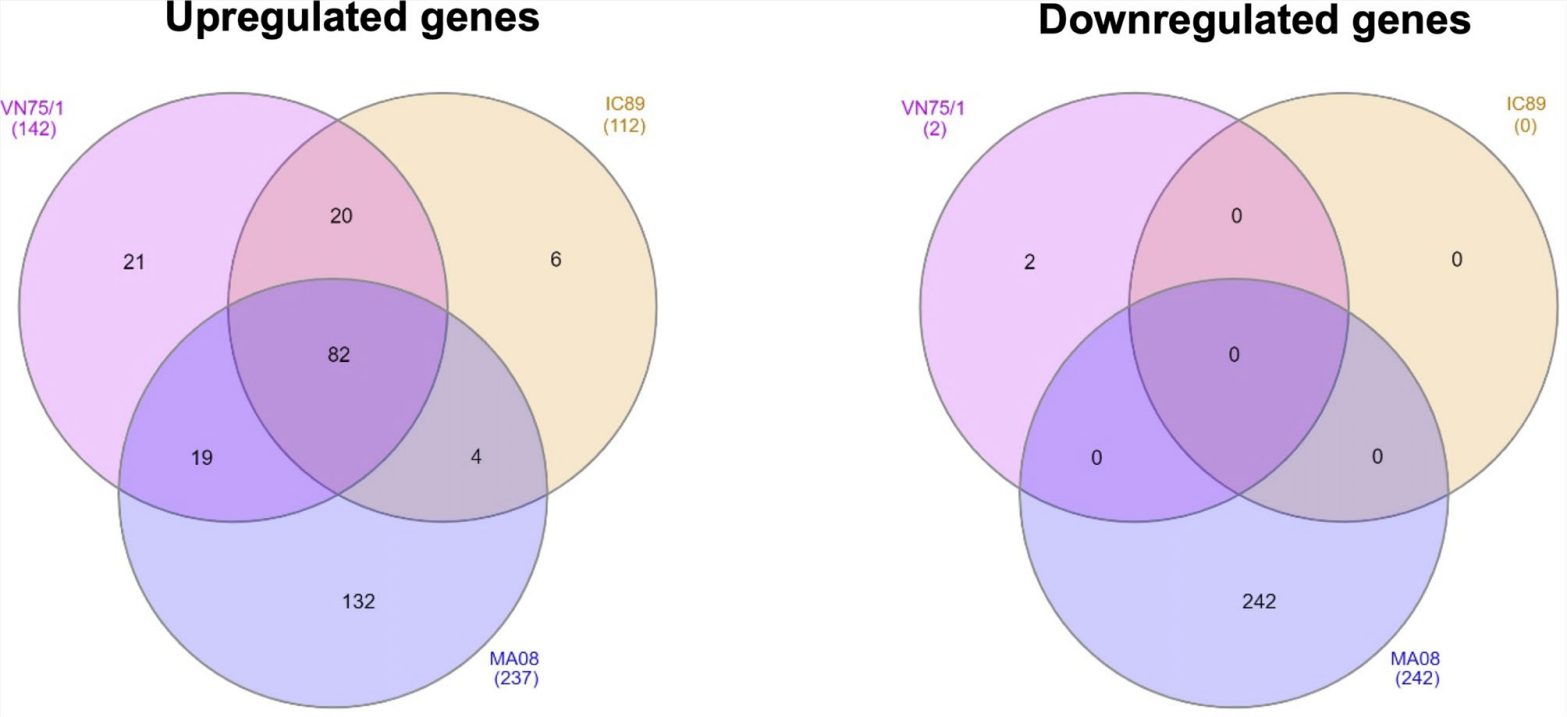
Venn diagrams of up- and down-regulated genes between PPRV infections determined using DESeq2. Venn diagrams were obtained using InteractiVenn. The gene lists were obtained by comparison with DESeq2 of each infection and the mock control (mock-infected cells) at 72 hpi. A threshold of |Log2FoldChange| >1 and an adjusted p-value < 0.05 were set.

Principal component analysis of the transcriptomic data at 72 hpi revealed distinct clusters that allowed further analysis. In this analysis, only the MA08 group was clearly distinguished from the IC89 and VN75/1 groups and the control group (**Supplementary Figure 1A**). Infections of PBMCs with PPRV strains induced up and downregulation of genes compared to mock-infected cells. Analysis of mRNA expression at 72 hpi showed that VN751 and IC89 strains mainly induced gene upregulation while MA08 induced both gene up- and downregulation. A total of 82 upregulated genes were common to the three infection groups, while 20 genes were specific to VN751 and IC89, and 19 genes were specific to VN751 and MA08. In addition, MA08 strain induced upregulation of 132 genes and downregulation of 242 genes not observed in other groups (**Figure 3**). The upregulated genes identified in the annotated reference genome and common to all treatment groups show similar expression levels. Categories retrieved from gene ontology show that most of commonly expressed genes are involved in immune processes such as the type I interferon-mediated antiviral response (**Supplementary Figure 2A**). Complementary genes such as those encoding RNASEL, TDRD7, UBA7 and UBE2L6 of this antiviral response are activated in common between IC89 and VN75/1 infections (**Supplementary Figure 2D**). Several non-coding RNA (7SK, Metazoa_SRP, U1 and 5_8S_rRNA) involved in the transcription mechanisms are expressed in common between the IC89, MA08 and MA08, VN751 infections (**Supplementary Figures 2E, F**). Genes involved in endoplasmic stress response, immune response, gene transcription and cytokine and chemokine activity are specifically upregulated upon infection with MA08 (**Supplementary Figure 2B**) while those involved in biosynthesis processes, development and maintenance of cell shape and function are downregulated (**Supplementary Figure 2C**).

### PPRV Infection of Cells Induces Differential Expression of Antiviral Genes and Proteins

**Figure 4:**
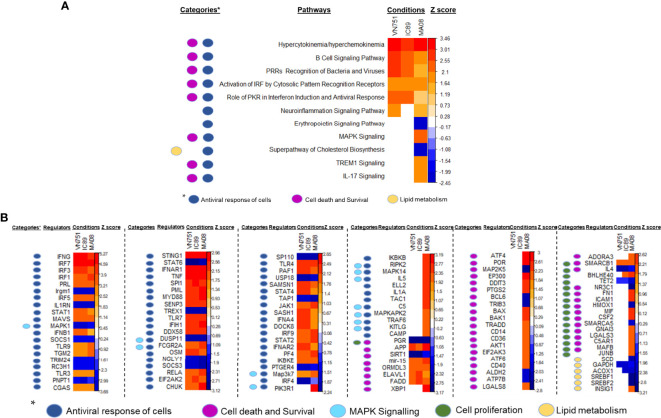
Identification of pathways and upstream regulators modified by PPRV infections at 72 hpi. Qiagen’s IPA software provided a Zscore that informs on the activation status of pathways **(A)** and upstream regulators **(B)**. Positive zscores (upregulated) are represented by an orange gradient while negative zscores (downregulated) are represented by a blue gradient. Pathways and upstream regulators have been classified in categories related to antiviral cell responses (dark blue square), MAPK signalling (light blue square), lipid metabolism (yellow square), cell death, survival (violet square) and proliferation (green square).

**Figure 5:**
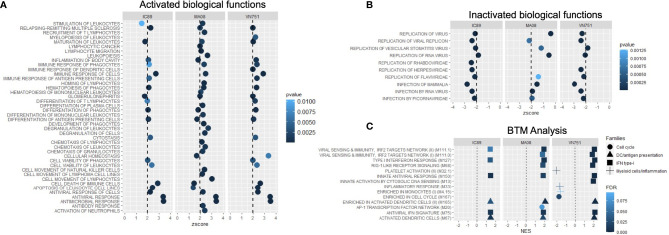
Analysis of the activation of biofunctions and BTMs during PPRV infections at 72 hpi. Activated **(A)** and inactivated **(B)** biofunctions were found during the analysis. Qiagen’s IPA software provided a zscore that determines the activation (zscore>2) and the inactivation (zscore<-2) status of biological functions. All inactivated functions represent virus infection and replication functions and therefore reflect the induction of antiviral activities in the cells. **(C)** Analysis of goat blood transcriptional modules (BTMs) during PPRV infections of PBMCs at 72 hpi. Goat BTMs were activated or inactivated during infections (significance level: FDR < 0.1). Positive normalised enrichment score (NES > 0) means activation of a set of genes while negative NES (NES < 0) means inactivation.

DEG lists were used to identify pathways, functions and regulators that are dysregulated in the different infection groups. Production of cytokines and chemokines, PRR recognition of virus, IRF activation and the role of PKR in antiviral pathways were upregulated in all conditions. Specifically, upregulation of MAPK TREM1 and IL17 signalling pathways and downregulation of erythropoietin and cholesterol biosynthesis pathways were observed after infection with MA08. These pathways have been classified as belonging to the antiviral response, cell death and survival and/or lipid metabolism (**Figure 4A**). More upstream regulators were also observed after infection with MA08 than with the other strains. Like the pathways, these regulators have been classified for their potential role in antiviral response, cell death and survival, MAPK signalling, cell proliferation and/or lipid metabolism (**Figure 4B**). Biological functions involved in antiviral response, differentiation and apoptosis of immune cells (lymphocytes and phagocytes) were activated in all infection groups. VN751 and IC89 activated similar functions. MA08 also activated several other functions. These specific functions were mainly related to the formation of lymphocytes and natural killer cells, migration, recruitment and inflammatory processes such as cell degranulation (**Figure 5A**). Biological functions involved in replication of the virus were significantly repressed across infections, particularly VN751 and IC89 strains that have a greater number of functions with a zscore lower than -2 (**Figure 5B**). Genes involved in this mechanism are shown in **Supplementary Figure 3**. Gene set enrichment analysis detected activated blood transcriptional modules (BTMs) involved in the antiviral type I interferon response and in antigen presentation by dendritic cells. BTMs involved in the role of myeloid cells in inflammation were found to be repressed upon infection with VN751 (**Figure 5C**).

**Figure 6:**
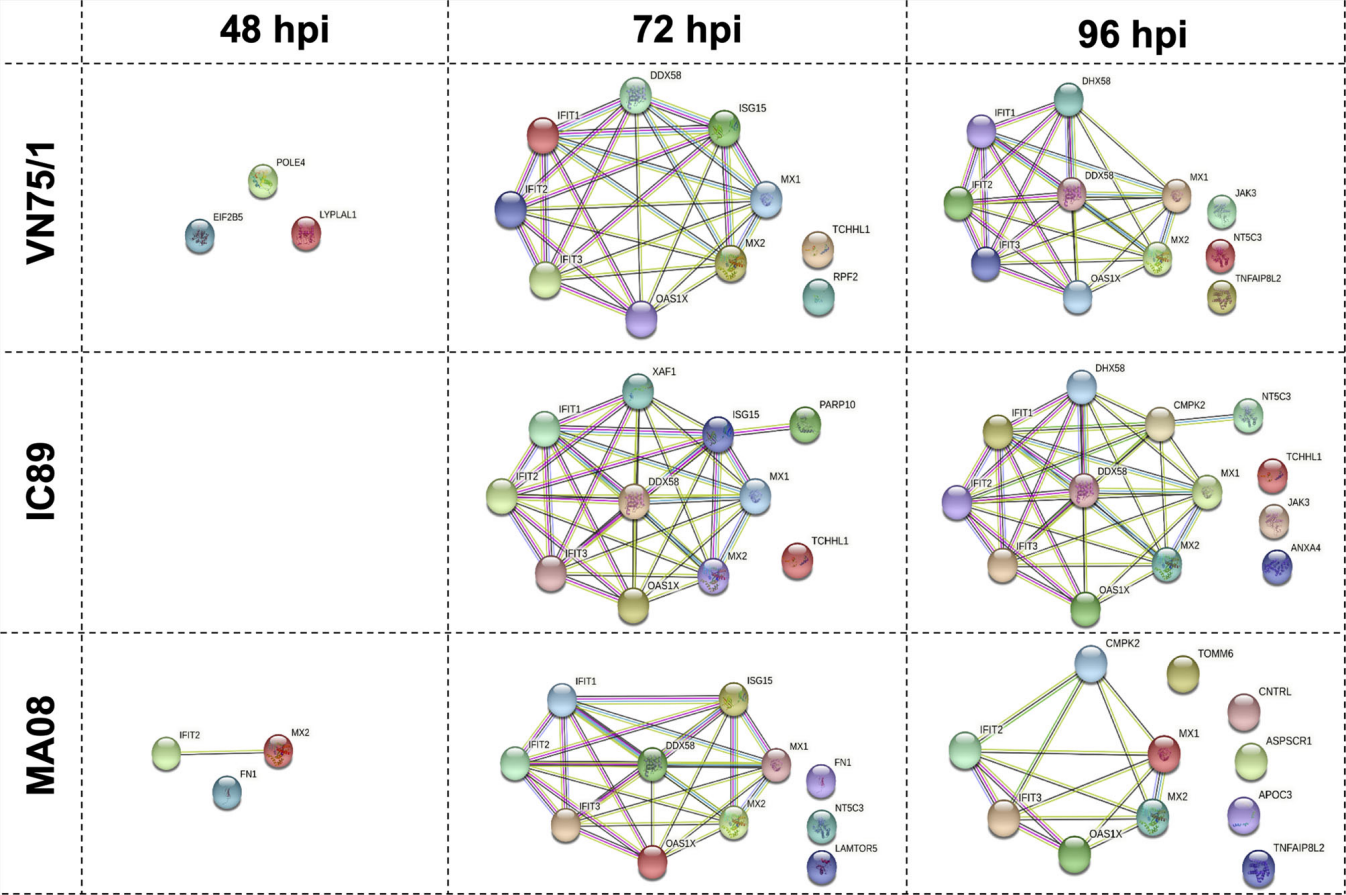
Protein-protein networks generated during PPRV infections of PBMCs. Data at 48, 72 and 96 hpi were obtained by comparing each infection (VN751, IC89, MA08) to the control (Mock infected cells). Each protein is represented by a different colour circle, picked randomly by the software. The colour of a same protein may differ between each analysis. The colours of the lines between the proteins determine the level of interactions. Known interactions are represented by pink (experimentally determined) and blue (from curated databases) lines. Green (text mining), black (co-expression) and grey (protein homology) lines represent other interactions.

Proteins differentially expressed between infections were analysed using a proteomic approach. Principal component analysis of the proteomic data at 48, 72 and 96 hpi revealed distinct clusters that reflect the course of the infections. The IC89 and VN751 groups had similar protein distribution and expression, while the MOCK and MA08 groups showed distinctive patterns (**Supplementary Figure 1B**). A set of proteins involved in the antiviral response was differentially expressed among the infection groups from 72 to 96 hpi (**Figure 6**). These proteins were significantly detected in all infections but at different expression levels. Fewer proteins were detected in the MA08 infection group. Some proteins identified at 72 hpi were no longer detected at 96 hpi, e.g., ISG15 in all conditions, IFIT1 and DDX58 (RIG I) in the MA08 group only. In addition, some proteins were detected only in specific infection groups: EIF2B5, POLE4 and LYPLA1 at 48 hpi, TCHHL1, RPF2 at 72 hpi and JAK3, NT5C3 and TNFAIP8L2 at 96 hpi for VN75/1; TCHHL1, PARP10 at 72 hpi and TCHHL1, JAK3 and ANXA4 at 96 hpi for IC89; FN1, MX2 and IFIT2 at 48 hpi, FN1, NT5C3 and LAMTOR5 at 72 hpi and TOMM6, CNTRL, ASPSCR1, APOC3 and TNFAIP8L2 at 96 hpi for MA08 (**Figure 6**).

## Discussion

The virulence of PPR viral strains depends on the host, the environment and the intrinsic abilities of the strains to replicate in infected cells. Host susceptibility has already been described in several in-vivo studies (17, 45) but the host factors underlying the differences in virulence of the viral strains are not yet clearly understood. In this study, an in-vitro approach was used to explore the mechanisms behind the different levels of virulence of three PPRV strains in Saanen goat PBMCs.

A very low MOI was used to monitor viral replication and its effect on lymphocyte survival and proliferation. After infection, PBMCs were stimulated with ConA, allowing increased surface expression of SLAM receptors and higher infectivity of PPRV strains (35, 46). In this context, the highly virulent Morocco 2008 (MA08) strain had a higher level of replication than the vaccine Nigeria 75/1 (VN75/1) and Ivory Coast 1989 (IC89) strains. In addition, all the strains used in this study had a negative impact on cell viability and inhibited lymphocyte proliferation, and the effect was earlier and stronger when PBMCs were infected with the MA08 strain. The inhibition of lymphocyte proliferation was concluded from a significantly higher level of undivided cells compared to that in the control group. Taken together, these results indicate that, in this cellular context, the highly virulent strain MA08 was able to replicate better and rapidly induce cell death and inhibit lymphocyte proliferation. Vaccine Nigeria 75/1 (VN75/1) and Ivory Coast 1989 (IC89) replicated more slowly and had a delayed inhibitory effect on lymphocyte proliferation. Therefore, this model was effective in distinguishing between highly and weakly virulent PPRV strains and suggests that the death of infected cells may play an important role in the *in vitro* inhibition of lymphocytes proliferation by PPRV. Similar behaviour has been observed in other *Morbilliviruses*. Measles virus-induced immunosuppression is caused by direct killing of infected memory T and B lymphocytes and indirect mechanisms such as deregulation of cytokines or surface contact-mediated signalling, which may lead to apoptosis or alter the proliferative response of uninfected PBMCs (47).

RNA sequencing and proteomic approaches were used to investigate the host cellular factors associated with the difference in replication between strains in stimulated PBMCs. Differentially expressed genes (DEGs) were widely present upon infection with the highly virulent MA08 strain, whereas VN75/1 and IC89 induced milder gene regulation and almost no down-regulation at 72 hpi. With respect to the upregulated genes expressed in a common way between strains, most of them appear to be IFN antiviral response genes that are induced at a similar level between infections. Previous studies have also shown that these interferon-stimulated genes (ISGs) are upregulated in the early stages of PPRV infection (25, 26). In addition, these ISGs help to strengthen host defence and reduce PPRV replication in goats and cattle (48).

Some upregulated genes were common to the VN751 and IC89 infection groups, but not expressed in MA08 infection. This small set of genes include genes such as HAVCR2, TDRD7, UBA7, UBE2L6 and RNASEL involved in altering viral replication. HAVCR2, also known as TIM3, is known to induce downregulation of cytokine production, in particular IL2, by T cells, and to suppress NFAT dephosphorylation (49). The interaction between galectin-9 and TIM3 expressed on the surface of activated CD4+ T cells induce resistance of activated CD4+ T cells to HIV-1 infection and replication (50). TDRD7 (Tudor Domain-containing 7) is a novel antiviral ISG expressed at low endogenous levels in various cell types and transcriptionally upregulated upon viral infection or IFN treatment. TDRD7 is antiviral by inhibiting the cellular autophagy pathway, which is required for paramyxo-/pneumovirus replication (51). UBA7 and UBE2L6 are essential enzymes for ISG15 conjugation (ISGylation). ISGylation plays an important role in establishing the antiviral state of infected cells (52). RNASEL (Ribonuclease L) limits viral infections by degrading viral and cellular RNA, inducing autophagy and apoptosis, and producing RNA degradation products that enhance type I interferon (IFN) production *via* RIG-I receptors (53). The regulatory status of genes involved in RNASEL synthesis, such as PDE12 (a negative regulator of the innate immune response), could be a major factor in resistance to PPRV replication (48). Taken together, our results and observations in the literature confirm that PPRV infections induce an antiviral state characterised by the induction of an antiviral IFN response. Other mechanisms involved in the inhibition of viral replication are induced by the activation of specific ISGs during infections with IC89 and VN75/1 strains. These mechanisms may explain the lower level of replication observed when these strains are used to infect PBMCs. A higher number of DEGs was found in the infection with the MA08 strain. This stronger regulation may be due to the higher replication capacity of this strain, which induces greater alteration in cell metabolism. This response at 72 hpi also correlates with the beginning of the decrease in cell viability and the inhibition of cell proliferation induced by this strain.

DEGs lists were used to analyse pathways and regulators involved in the response of cells to infection with PPRV strains of different virulence. Production of large amount of cytokines (hypercytokemia/hyperchemokinemia), B cell signalling, pathogen recognition receptors (PRRs) recognition of viruses, activation of interferon regulatory factors (IRF) by cytosolic PRRs (MDA-5, RIG-I and LGP2) and the role of protein kinase R (PKR) in interferon induction were found to be upregulated in all infection groups at 72 hpi. These pathways, involved in the induction of the antiviral state and cell death, further confirmed the common effect on PBMCs generated by PPRV infections. BTM analysis of the lists of ranked DEGs at 72 hpi also confirmed the involvement of type I interferon modules in the response to infection by all PPRV strains. Overall, the genes involved in these pathways and in BTMs seemed to orchestrate a classical antiviral response regardless of the virulence of the PPRV strain. In addition, specific upregulation of the MAPK, TREM1 and IL17 signalling pathways was induced after infection with MA08. These specific pathways are part of the cellular pro-inflammatory and antiviral response induced by this strain (54, 55). These specific responses were also accompanied by downregulation of erythropoietin (EPO) signalling pathways and cholesterol biosynthesis. Therefore, our results suggest that the highly virulent MA08 strain is capable of inducing a more diverse response of cells. For instance, cell death could be indirectly activated by the inhibition of the anti-apoptotic EPO signalling (56). This strain could also have an impact on cellular metabolism by altering cholesterol biosynthesis, a complementary mechanism that enhances the antiviral response of cells (57). BTM analysis succeed in the detection of the main induced response of infected cells and was also able to detect inactivation of certain modules involved in the role of myeloid cells in inflammation, dendritic cell activation and in the cell cycle. Thus, this tool has confirmed its effectiveness and could be used in the future to detect the mechanism of action and factors related to the response of veterinary species to infection or vaccination (38).

Upstream regulators are genes or transcriptional regulators that govern multiple pathways and gene activation. These regulators may explain the observed changes in gene expression and pathway activation. Upstream regulator analysis is based on knowledge of the expected effects between transcriptional regulators and their target genes stored in the Ingenuity knowledge database. Since the activation zscore of these regulators is obtained by a calculation based on the presence and activation status of their known targets, it is not surprising to have inactivated upstream regulators although there are no downregulated genes in the data set (58). Categories extracted from the IPA analysis showed that these regulators are involved in the activation of the antiviral response of cells, cell death and proliferation, MAPK signalling and lipid metabolism. The activation status of these regulators is consistent with the pathway’s activation status. For instance, the inactivation of the cholesterol biosynthetic super-pathway must be governed by the downregulation of GAPDH, ACOX1, SREBF1/2 and the upregulation of INSIG1. INSIG1 is an important upstream regulator that effectively controls the transcription of SREBF elements preventing the activation of lipid biosynthesis genes (59). Upstream regulators involved in the control of cell proliferation are specifically activated upon infection with PPRV MA08 strain. These regulators have typically double-edged sword abilities, as they are involved in both cell proliferation and cell death and survival. Taken together, these results suggest that the induction of the antiviral state is governed by the activation of several upstream regulators. These regulators could have many effects on cellular responses and, therefore, these experiments provide some specific key targets to study that could be involved in the cell division inhibition induced by PPRV infection.

Many of them had the same regulatory status in all the conditions, but some of them were specific to the MA08 infection. For instance, the upregulation of MAPK14 and MAPKAAPK2 could explain the upregulation of MAPK signalling pathways observed after infection with MA08.

Several biological functions were regulated in similar ways in all infection groups at 72 hpi, with a higher number of functions activated after infection with MA08. These functions included antiviral response, differentiation and apoptosis of immune cells (lymphocytes and phagocytes). VN751 and IC89 have mainly activated similar functions, while MA08 induced the activation of several other functions. These specific functions are related to cell formation and migration (lymphocytes and natural killer cells) and granule cell degranulation. In addition, the biological functions involved in virus replication were significantly repressed during infections, especially VN751 and IC89 strains.

Genes specifically involved in the repression of viral replication such as HAVCR2, TDRD7, UBA7, UBE2L6 and RNASEL were first found in the comparison of DEGs between the two infections VN751 and IC89. The repression of biofunctions related to viral replication thus confirmed the previous analysis and furthermore suggests the potential involvement of these mechanisms in the virulence of a PPRV strain. These genes were upregulated in VN751 and IC89 infections, while not in MA08 infection. For instance, HAVCR2 involved in NFAT dephosphorylation (49) was not significantly regulated in MA08 infection and was accompanied by upregulation of IL2 and downregulation of NFATC2 (nuclear factor of activated T cells, cytoplasmic 2). The downregulation of one of the cytoplasmic form of NFAT in the MA08 infection (**Supplementary Figure 3**) suggests dephosphorylation and translocation of this factor into the nucleus. AGO4, SNX9, SCD were also downregulated after infection with MA08. Decreased expression of AGO4 (Argonaute 4) leads to a reduction of replication in RSV infection (60), but a contradictory role for this gene was found *in vivo* in mice infected with the influenza virus. AGO4 deficiency in influenza infection results in higher viral titres *in vivo* (61). The opposing effects of decreased expression of this gene may raise questions about its pro- or antiviral role in PPRV infection. Downregulation of SNX9 (Sorting nexin-9) is associated with a state of chronic inflammation and immunosuppression and its monitoring, together with that of CD247 expression, could serve as an “optimal sensing” tool to define the severity of chronic inflammation and induced immunosuppression (62). SCD1 (Stearoly-CoA desaturase 1) inhibition disrupts virus replication and induces the production of non-infectious virus particles (63, 64). Taken together, these results may indicate that the high replication rate of the highly virulent PPRV MA08 strain may be due to the involvement of certain viral proteins that interact directly with the activation of certain antiviral gene such as HAVCR2 and RNASEL. However, complementary mechanisms seemed to be induced to compensate for the absence of these genes. SNX9 could be an in-vitro marker for the interruption of mitogen-induced immunosuppression by PPRV.

A proteomic analysis was performed in an attempt to link gene and protein expression. As observed in the transcriptomic data, the proteins involved in the type I interferon antiviral response were observed in all infections. Clear kinetics of this response were observed during infection with MA08, starting with the production of ISGs, IFIT2 and MX2 at 48 hpi. The induction of interferon antiviral molecules has already been observed at earliest infection stages (26, 65). Then, the results of our study confirmed that interferon stimulated elements are not only transcriptionally but also translationally activated in the early stages of infection.

A core of interferon-related proteins was detected in all infections at 72 hpi, although expression of XAF1 and PARP10 was also detected only after infection with IC89. At 96 hpi, overall expression of proteins belonging to the core interferon-related proteins was similar in IC89 and VN75/1 infections. At this stage, fewer proteins were detected after infection with MA08. This could be due to the exhaustion of the cells, which started to react at 48 hpi. Some specific proteins also appeared at different stages of the infections. For example, after infection with MA08, these were proteins such as CNTRL involved in cell cycle progression and the late stages of cytokinesis, TNFAIP8L2, involved in the different stages of chronic viral infection, and LAMTOR5, are essential for the coordination of nutrient and inflammatory signals to shape optimised immune response (66–68). These proteins may play a role in the phenotype observed upon infection with this strain. Some other specific proteins such as JAK3 were expressed in IC89 and VN75/1 infections. JAK3 is a well-known protein involved in JAK/STAT pathway which plays a major role in cytokine receptor signalling and IFN response (69).

**Figure 7:**
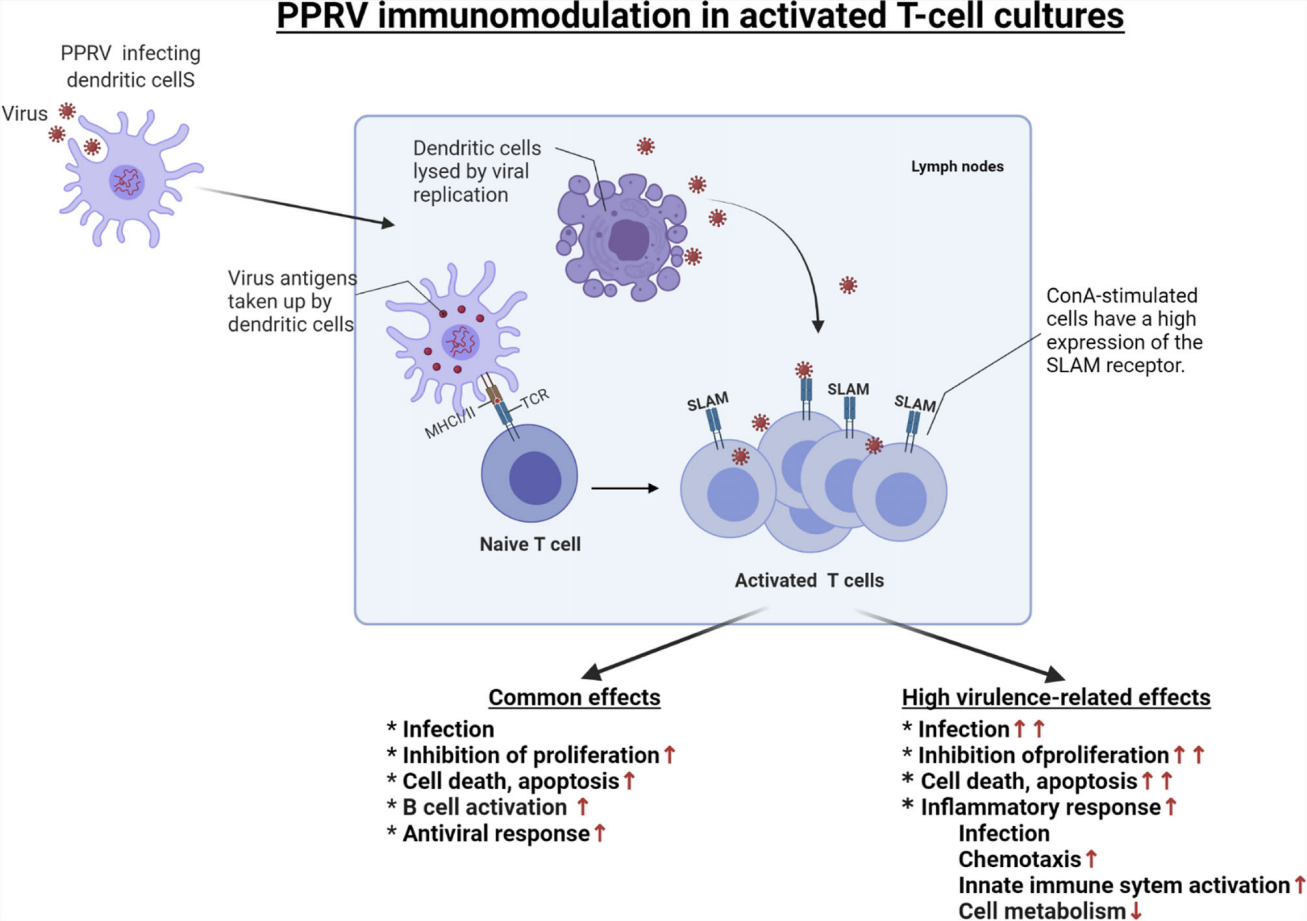
Representation of the mechanisms involved in the infection of activated PBMCs by different virulence strains of PPRV. The first steps of PPRV infectioninclude the transport of the virus by antigen-presenting cells (represented by dendritic cells) from the site of infection to the lymph nodes. Once in the lymph nodes, these cells trigger the activation and proliferation of T cells. This activation is associated with the expression of the SLAM receptor, as observed upon stimulation byConA. The virus may exit the DCs and then encounter the activated cells. The common effects of PPRV strains upon infection of activated T cells are the activation of an antiviral response, the inhibition of cell proliferation and the induction of cell death and apoptosis. In the case of a highly virulent strain, these activated cells are further infected and the inflammatory response is greater. Created with BioRender.com.

Finally, upregulation of key antiviral genes detected by RNA sequencing was confirmed by their translation using proteomic analysis. Both transcriptomic and proteomic approaches were successful in analysing the antiviral response of cells. However, proteins involved in stress responses and other mechanisms were not significantly detected in the proteomics-based approach. Multilevel analyses of cellular response to infection with PPRV strains of different virulence provided insights into the factors underlying the observed phenotypes. In the model used in this study, the Nigeria 75/1 vaccine and the weakly virulent Ivory Coast 1989 strain acted broadly similarly during infection. Both strains replicated slowly but were able to inhibit cell proliferation and induce activation of the antiviral response and cell death. The induction of specific genes such as HAVCR2, TDRD7, UBA7, UBE2L6 and RNASEL may be associated with the lower replication capacity of these strains. The highly virulent Morocco 2008 strain replicated more rapidly and acted earlier on cell metabolism by more strongly inhibiting proliferation, inducing antiviral responses and apoptosis using several means. Its high level of replication could be partially explained by mechanisms inhibiting activation of the same genes. The lack of activation of these genes seems to be compensated by the induction of other complementary mechanisms that are insufficient to inhibit viral replication of this strain. A summary of the mechanisms involved in this *in vitro* PPRV infection of activated PBMCs is shown in **Figure 7**.

These in-vitro results provide only a partial explanation for the variability observed in the field. To gain further knowledge, this type of analysis should be carried out in an in-vivo model to confirm the trends we observed and to identify other factors that could explain the variability in virulence. This kind of research, repeated to other virus strains and host species, could provide a better understanding of the factors underlying the spread of the disease and help to improve surveillance and control of peste des petits ruminants.

## Data Availability Statement

The datasets presented in this study can be found in online repositories. The RNA sequencing raw data have been deposited in the Sequence Read Archive (SRA) under the following identifier PRJNA748573. The mass spectrometry proteomics data have been deposited in the ProteomeXchange Consortium *via* the PRIDE (70) partner repository with the dataset identifier PXD024890 and 10.6019/PXD024890.

## Ethics Statement

Blood sampling was performed in compliance with the Swiss animal protection law (TSchG SR 455; TSchV SR 455.1; TVV SR 455.163) under the authorization number BE8/19. The experiments were reviewed by the cantonal committee on animal experiments of the canton of Bern, Switzerland, and approved by the cantonal veterinary authority (Amt für Landwirtschaft und Natur LANAT, Veterinärdienst VeD, Bern, Switzerland).

## Author Contributions

Project conceptualization, R-JE, GA, VR, AB, AS. Data acquisition and analysis, R-JE, GA, SP, MS, SU, KE, PH, and AS. Funding acquisition and investigation, GL, AB, and AS. Drafting of the manuscript, R-JE. Manuscript review and editing, R-JE, VR, MS, PT, GL, AB, and AS. Project administration and supervision, AB and AS. All authors contributed to the article and approved the submitted version.

## Funding

This research was co-funded by the European Commission through the VetBioNet (grant no. 731014) and was supported by a grant (SI2.756606) from the European Commission Directorate General for Health and Food Safety awarded to the European Union Reference Laboratory for Peste des Petits Ruminants (EURL-PPR).

## Conflict of Interest

The authors declare that the research was conducted in the absence of any commercial or financial relationships that could be construed as a potential conflict of interest.

## Publisher’s Note

All claims expressed in this article are solely those of the authors and do not necessarily represent those of their affiliated organizations, or those of the publisher, the editors and the reviewers. Any product that may be evaluated in this article, or claim that may be made by its manufacturer, is not guaranteed or endorsed by the publisher.

## Supplementary Material

Supplementary Figure 1 Principal component analysis (PCA) of the RNA sequencing and proteomic data collected from PPRV infections of PBMCs. **(A)** PCA of RNA sequencing data obtained in each condition at 72 hpi. **(B)** PCA of proteomic data obtained in each condition at 48 hpi (J2), 72 hpi (J3) and 96 hpi (J4). CTRL in proteomic data represent the Mock group.

Supplementary Figure 2 Common and specific regulated genes between PPRV infections. **(A)** Gene ontology (GO) analysis of commonly upregulated genes between all infections. **(B)** GO analysis of specifically upregulated and downregulated genes **(C)** in MA08 infection. Source represents significant (adjusted pvalue < 0.05) GO categories BP (biological processes), CC (Cellular Components), MF (Molecular Functions) and KEGG pathways obtained from g:Profiler. The rich factor is the number of genes found in the analysis divided by the number of genes in the GO category. **(D–F)** represent the log2 fold change values of upregulated genes found in common between VN75/1 and IC89 **(D)**, MA08 and VN75/1 **(E)** and VN75/1 and IC89 infections.

Supplementary Figure 3 Representation of genes involved in the repression of viral replication during PPRV infections. Red circles, squares, triangles or diamonds represent genes that are upregulated during infection, while green ones represent genes that are downregulated. Genes found several times in the analysis are tagged with an asterisk. The broken lines represent the predicted relationships between genes and biofunction. Blue lines lead to inhibition, yellow lines represent results inconsistent with the state of the downstream molecule and grey lines represent non-predicted effects.
