# Supplementary figures and images for: Identification of Differential Responses of Goat PBMCs to PPRV Virulence Using a Multi-Omics Approach
Source: Front Immunol. 2021 Oct 4;12:745315. doi: 10.3389/fimmu.2021.745315 (PMC8521192; doi:10.3389/fimmu.2021.745315)

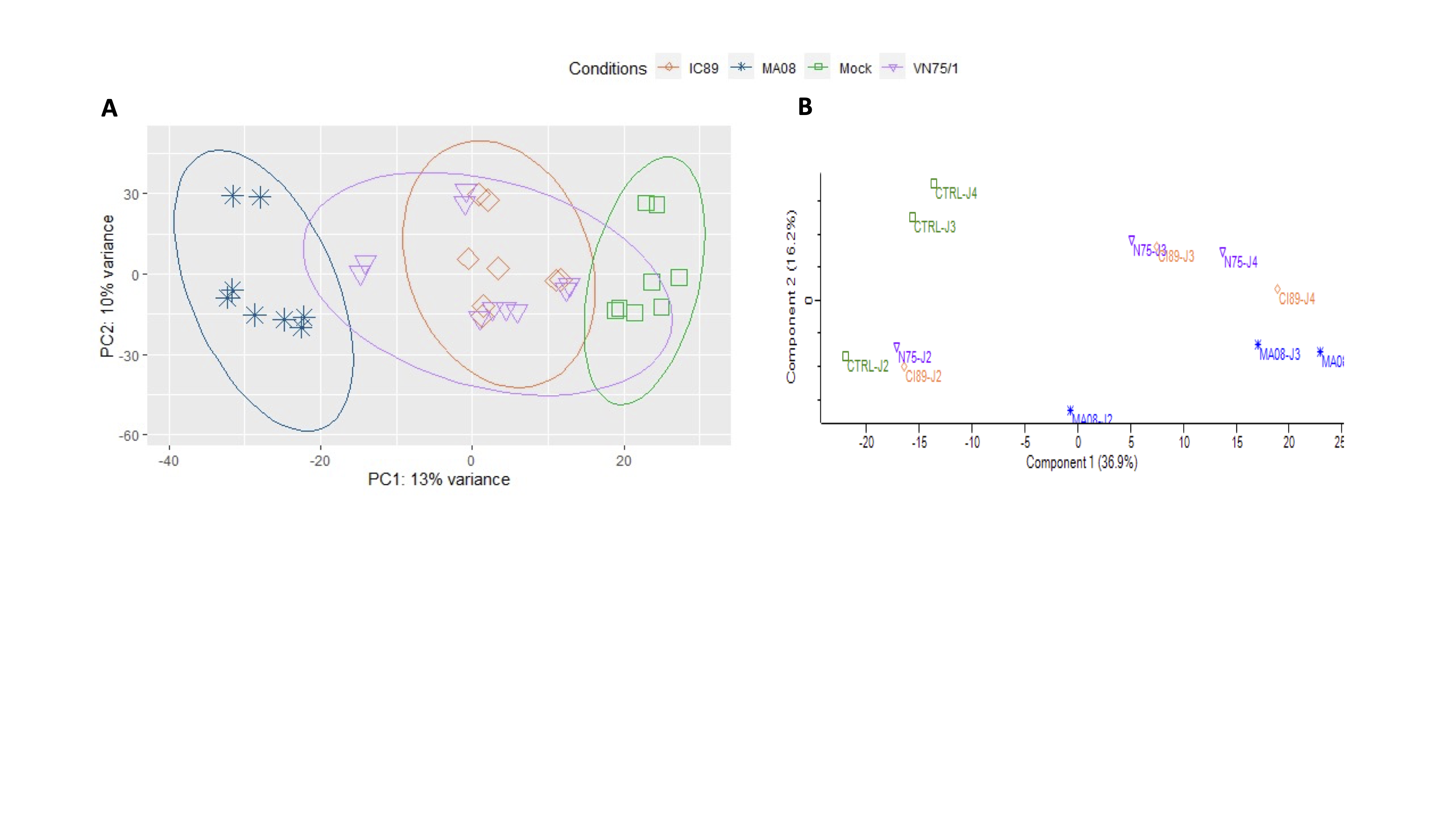

Supplement: Supplementary Figure 1 — Principal component analysis (PCA) of the RNA sequencing and proteomic data collected from PPRV infections of PBMCs. (A) PCA of RNA sequencing data obtained in each condition at 72 hpi. (B) PCA of proteomic data obtained in each condition at 48 hpi (J2), 72 hpi (J3) and 96 hpi (J4). CTRL in proteomic data represent the Mock group. [file Image_1.tiff]

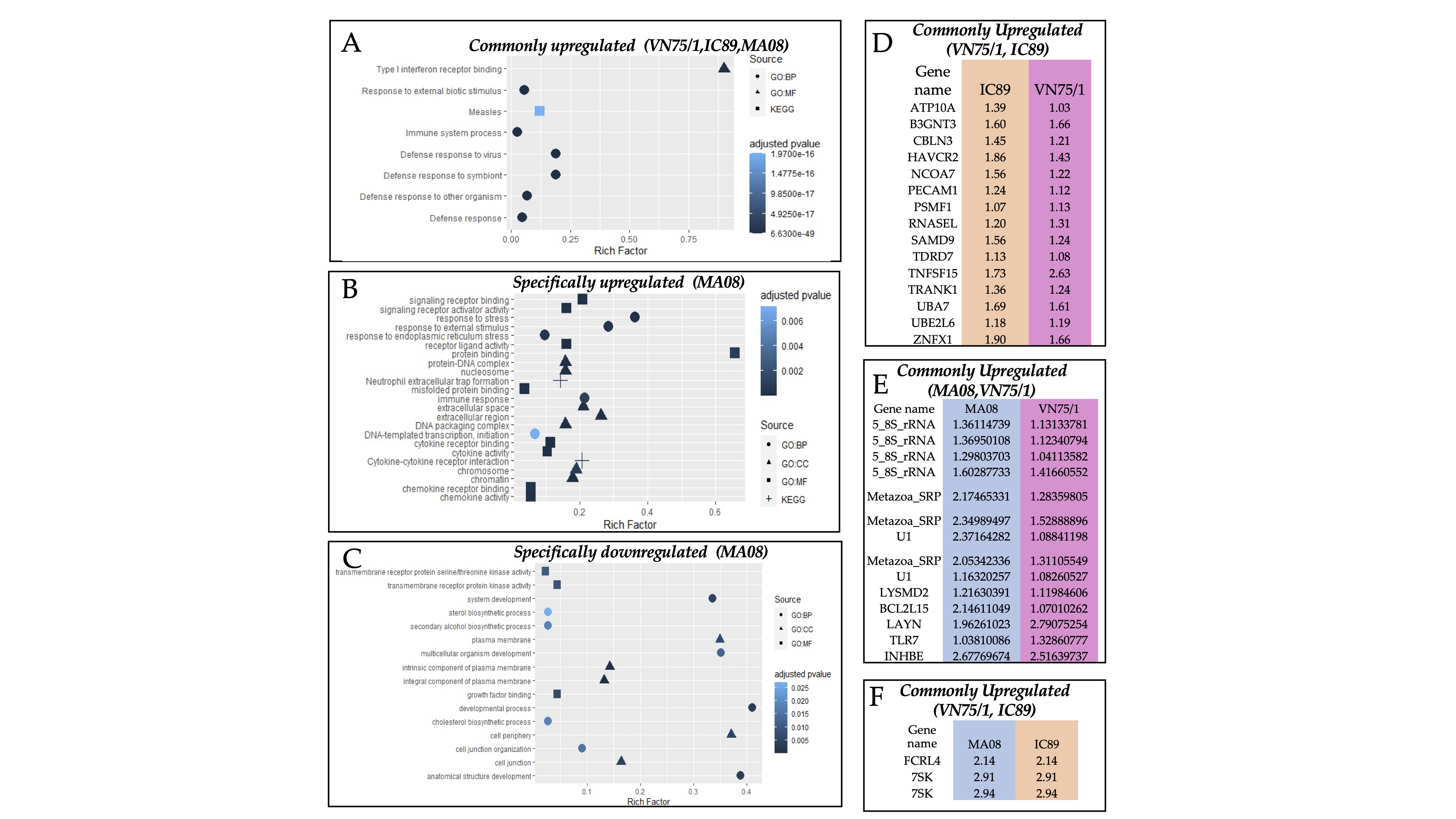

Supplement: Supplementary Figure 2 — Common and specific regulated genes between PPRV infections. (A) Gene ontology (GO) analysis of commonly upregulated genes between all infections. (B) GO analysis of specifically upregulated and downregulated genes (C) in MA08 infection. Source represents significant (adjusted pvalue < 0.05) GO categories BP (biological processes), CC (Cellular Components), MF (Molecular Functions) and KEGG pathways obtained from g:Profiler. The rich factor is the number of genes found in the analysis divided by the number of genes in the GO category. (D–F) represent the log2 fold change values of upregulated genes found in common between VN75/1 and IC89 (D), MA08 and VN75/1 (E) and VN75/1 and IC89 infections. [file Image_2.tiff]

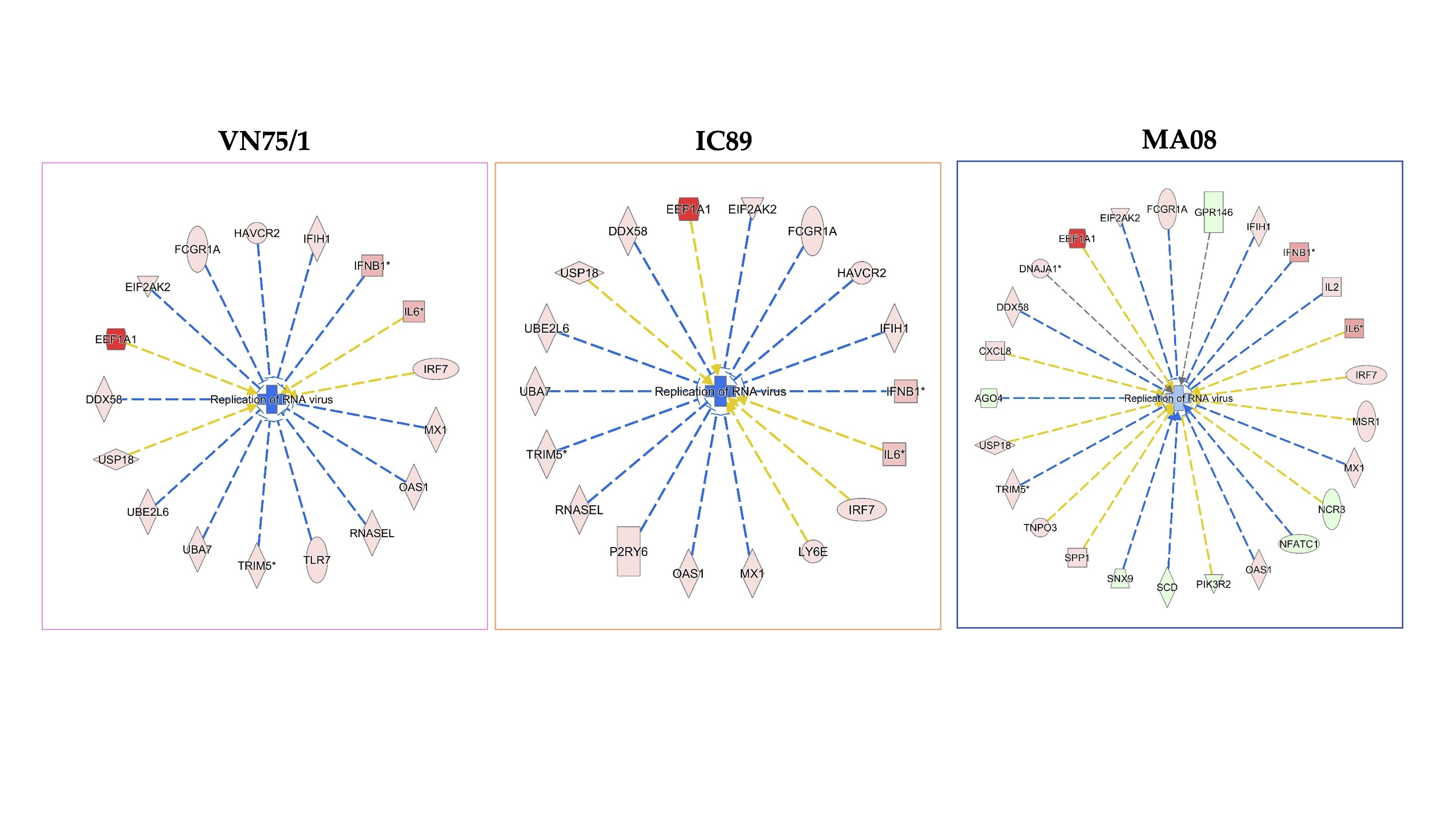

Supplement: Supplementary Figure 3 — Representation of genes involved in the repression of viral replication during PPRV infections. Red circles, squares, triangles or diamonds represent genes that are upregulated during infection, while green ones represent genes that are downregulated. Genes found several times in the analysis are tagged with an asterisk. The broken lines represent the predicted relationships between genes and biofunction. Blue lines lead to inhibition, yellow lines represent results inconsistent with the state of the downstream molecule and grey lines represent non-predicted effects. [file Image_3.tiff]
